# Chronic Limb‐Threatening Ischemia Shows No Adaptability of Mitochondrial Function After Revascularisation

**DOI:** 10.1111/jcmm.71383

**Published:** 2026-10-16

**Authors:** Fiona Speichinger, Alexandra Gratl, Ben Raude, Marco Arndt, Nina A. Hering, Andreas Greiner, Dominik Pesta, Christian Schineis, Jan Paul Frese

**Affiliations:** ^1^ Department of General and Visceral Surgery Charité Universitätsmedizin Berlin Berlin Germany; ^2^ Department of Vascular Surgery Charité Universitätsmedizin Berlin Berlin Germany; ^3^ Department of Vascular Surgery Konventhospital Barmherzige Brueder Linz Linz Austria; ^4^ German Aerospace Center Institute of Aerospace Medicine Cologne Germany; ^5^ Medical Faculty University of Cologne Cologne Germany; ^6^ Cologne Excellence Cluster on Cellular Stress Responses in Aging‐Associated Diseases (CECAD) University of Cologne Cologne Germany

**Keywords:** chronic limb threatening ischemia, intermittent claudication, mitochondrial function, mitochondrial respiration, myopathy, PAD, peripheral arterial disease, respiratory chain

## Abstract

Peripheral arterial disease (PAD) impairs limb perfusion, leading to myopathy characterised by altered mitochondrial content and respiration. Because ischemic muscle appears to adapt differently across PAD stages, we examined stage‐specific skeletal‐muscle mitochondrial function before and after successful revascularisation. Muscle biopsies of gastrocnemius and vastus lateralis muscle were obtained from 32 patients—21 with intermittent claudication (IC) and 11 with chronic limb‐threatening ischemia (CLTI)—before and 6 weeks after successful revascularisation. Mitochondrial respiration was assessed by high resolution respirometry (HRR). HRR results were normalised to citrate synthase activity (CSA) as a marker of mitochondrial content. Inter‐individual variation was expressed as the gastrocnemius‐to‐vastus respiration ratio (‘GV ratio’). In the IC group, CSA‐normalised mitochondrial respiration of gastrocnemius muscle was significantly reduced after revascularisation, paralleled by a significant decline in the GV ratio. In contrast, CLTI patients showed no significant pre‐ to postoperative change in CSA‐normalised respiration or GV ratio, but mitochondrial content significantly improved by revascularisation. Mitochondrial respiration 6 weeks after successful revascularisation adapts only modestly in milder PAD stages such as IC, while more advanced stages (CLTI) display no functional adaptations. These findings suggest that mitochondrial adaptive capacity is stage‐dependent and may require targeted therapeutic strategies beyond revascularisation alone, especially in CLTI.

**Abbreviations**
ABPI: ankle brachial pressure index
ADP: adenosine diphosphate
BIOPS: biopsy preservation solution
BMI: body mass index
CLTI: chronic limb threatening ischemia
CSA: citrate synthase activity
ET: electron transfer capacity
FCCP: carbonyl cyanide p‐(trifluoromethoxy) phenylhydrazone
GM: gastrocnemius muscle
GV: gastrocnemius muscle over vastus muscle
Hb: haemoglobin
HbA1c: glycated haemoglobin
HEPES: 4‐(2‐hydroxyethyl)‐1‐piperazineethanesulfonic acid
HRR: high‐resolution respirometry
IC: intermittent claudication
IQR: interquartile range
LC–MS/MS: liquid chromatography–tandem mass spectrometry
MiR06: mitochondrial respiration medium
N: NADH electron transfer‐pathway state
NADH: nicotinamidadenindinukleotid
No.: number
OXPHOS: oxidative phosphorylation
P: OXPHOS capacity
PAD: peripheral arterial disease
Rot: rotenone
S: succinate
SUIT: substrate‐uncoupler‐inhibitor titration
SVS WIfI: Society for Vascular Surgery Lower Extremity Threatened Limb classification system; wound, ischemia, and foot infection
tcPO_2_
: transcutaneous oxygen saturation
TMT: tandem mass tag
VLM: vastus lateralis muscle
vs.: versus

## 1 Introduction

Peripheral arterial disease (PAD) is one of the leading cardiovascular diseases worldwide, imposing a growing burden on healthcare systems [1, 2]. Clinically, PAD is stratified by severity. Milder stages, defined by intermittent claudication (IC), are characterised by exertional pain that resolves with rest, whereas the most severe stage of PAD, chronic limb threatening ischemia (CLTI), is defined by persistent rest pain, trophic lesions, < 0.4 ankle brachial index (ABPI), < 30 mmHg toe pressure, < 10 mmHg transcutaneous oxygen saturation (tcPO_2_) [3]. According to the Rutherford classification, IC corresponds to stages 2–3 and CLTI to Stages 4–6 [4].

Progression from IC to CLTI is driven by cardiovascular risk factors such as diabetes, hypertension, smoking and ageing [5]. Reported progression rates vary widely—1.1% to 36.7% over a mean follow‐up of 5.4 years—owing to differences in study design and definitions [5]. Pathophysiologically, PAD stages diverge at molecular, subcellular, and cellular levels. Arterial stenosis reduces skeletal muscle perfusion, limiting oxygen and nutrient delivery and fostering myopathy. Repeated ischemia–reperfusion cycles further impair cellular metabolism, intensifying mitochondriopathy and myopathy as the disease advances [6, 7, 8, 9]. Progressive fibrosis, altered gene expression, and distinct metabolic profiles can be observed in affected CLTI muscles compared with both IC and non‐PAD controls [10, 11]. Dysfunctional mitochondrial respiration elevates oxidative stress because antioxidant capacity is diminished; this can be quantified by Clark electrode measurements [12, 13] or by high‐resolution respirometry (HRR) [14]. Recent work has shown limited adaption of mitochondrial respiration in advanced PAD stages such as CLTI [15], whereas patients with isolated stenosis of the superficial femoral artery (SFA) and mild PAD exhibit adaptability of ischemic gastrocnemius muscle after revascularisation [16]. We hypothesise that adaptive capacity of the mitochondrial respiration after successful revascularisation is impaired in advanced PAD stages and address this in the present study.

## 2 Methods

### 2.1 Study Population

Consecutive patients presenting with symptomatic PAD who required treatment were screened at our outpatients' clinic for the following inclusion criteria: age > 18 years and elective conventional or endovascular revascularisation. Cardiovascular risk factors, current medication, and anthropometric data (body mass index (BMI), age, sex) were recorded. Diabetes mellitus was defined according to the criteria of the American Diabetes Association [17] as a fasting plasma glucose ≥ 126 (≥ 7.0 mmol/L) and/or HbA1c ≥ 6.5% (≥ 48 mmol/mol haemoglobin), irrespective of type. Surgical techniques of revascularisation were divided into open surgery, endovascular surgery and a combination of both the hybrid revascularisation. Before and 6 weeks after revascularisation every patient underwent a complete vascular examination including pulse status, ankle brachial pressure index (ABPI), and inspection for trophic lesions. Additionally, Doppler duplex ultrasound of the peripheral arteries to quantify and localise atherosclerotic stenoses (aorto‐iliac, femoro‐popliteal, distal), and a treadmill test (0% incline, 3 km/h) to determine maximum walking distance were performed. The endpoint was defined as a walking distance of 500 m without the onset of claudication. Depending on clinical severity of disease, patients were classified as Intermittent Claudication (IC; Rutherford 2–3) or Chronic Limb‐Threatening Ischemia (CLTI; Rutherford 4–5) [4] as well as according to the Society for Vascular Surgery Lower Extremity Threatened Limb (SVS WIfI) classification system [18]. The SVS WIfI is based on three main factors: wound, ischemia, and foot infection (WIfI) and is intended to classify the heterogeneous CLTI patient population more accurately in order to assess the risk of amputation and to guide clinical management. Each factor is divided into four levels ranging from 0 to 3 (none–mild–moderate–severe) [18]. However, non‐compressible ABPI measurements due to diabetes were excluded from the WIfI classification, as an ischemia grade 0 would incorrectly suggest the absence of ischemia. Six weeks after revascularisation, ABPI and walking distance were reassessed and restenosis was excluded by ultrasound.

### 2.2 Ethics

The study was approved by the Ethics Committee of Charité‐Universitätsmedizin Berlin (number of proposal EA4/139/16) and conducted in accordance with the Declaration of Helsinki and its latest 2013 amendment. All participants provided written informed consent.

### 2.3 Muscle Biopsy and Muscle Fibre Permeabilisation

Bergstrom Muscle Biopsy Needles (Ø 4.0 mm, Dixon Surgical Instruments, Wickford, UK) [19] were used for percutaneous muscle biopsies of vastus lateralis and gastrocnemius muscles of the affected leg under general or local anaesthesia; intramuscular lidocaine infiltration was avoided. Samples were collected perioperatively and again 6 weeks after revascularisation. Approximately 5 mg of muscle tissue was transferred into ice‐cold preservation medium (BIOPS; Supporting Information S1) and mechanically dissociated. Fibres were chemically permeabilised in BIOPS containing 50 μg/mL saponine for 30 min, then washed for 10 min in MIR06 mitochondrial respiration medium (Supporting Information S1). Muscle fibres (1–3 mg wet mass) were transferred immediately to the respirometry chamber, as described previously [14, 16, 20].

### 2.4 High‐Resolution Respirometry (HRR)

HRR was conducted using the Oxygraph‐2k (Orobros Instruments, Innsbruck, Austria) at a constant temperature of 37°C, following previously established protocols [14]. Each of the two respirometry chambers contained 2 mL MIR06 respiration medium. Oxygen levels and oxygen consumption rates were continuously monitored with polarographic sensors, and mass‐specific respiration was quantified as oxygen flux normalised to tissue mass, expressed in pmol of oxygen consumed per second per milligram of wet muscle tissue (pmol s^−1^ mg^−1^). To ensure sufficient oxygen supply and prevent diffusion limitation during measurement, chambers were first hyperoxygenated by addition of gaseous oxygen to achieve concentrations ~500 mM. A specific sequence of respiratory states was elicited by applying a substrate–uncoupler–inhibitor titration (SUIT) protocol, as outlined in prior studies [9, 14, 16]. LEAK state was induced by titration of malate (2 mM) and 0.2 mM octanoylcarnitine (MOct_L_). LEAK state represents mitochondrial respiration in absence of adenosine diphosphate (ADP). Subsequently, 5 mM ADP was introduced to initiate Complex I linked respiration (MOct_P_). Further Titration of 5 mM pyruvate (5 mM) and succinate (10 mM) activated combined Complex I and II driven oxidative phosphorylation (OXPHOS) capacity (P). Maximum electron transfer (ET) capacity was reached by stepwise titration of the uncoupling agent carbonyl cyanide p‐(trifluoromethoxy) phenylhydrazone (FCCP) in 0.05 mM steps, allowing for the assessment of the maximal respiratory chain activity. Addition of 0.5 μM rotenone as an inhibitor of complex I isolated respiration of Complex II [14].

### 2.5 Citrate Synthase Activity (CSA) Measurement

CSA was determined from frozen muscle specimens stored at −80°C. Upon thawing, tissues were transferred into CellLytic MT reagent (Sigma–Aldrich, St. Louis, MO, USA), mechanically homogenised and centrifuged. Total protein concentration in the supernatant was measured using the QuantiPro BCA Assay Kit (Sigma–Aldrich, St. Louis, MO, USA). CSA was then assessed spectrophotometrically at 412 nm and 25°C using the Citrate Synthase Assay Kit (Sigma–Aldrich, St. Louis, MO, USA). All mitochondrial respiration values obtained via HRR were normalised to CSA to account for differences in mitochondrial content.

### 2.6 GV Ratio

To allow for inter‐individual comparison, mitochondrial respiration rates and CSA values from the gastrocnemius muscle were compared to those from the vastus lateralis muscle. The resulting ratio (‘GV Ratio’) served as an internal reference, as previously described [15].

### 2.7 Statistics

Statistical analysis was performed using SPSS 27 (IBM, Armonk, NY). Because continuous data did not follow a Gaussian distribution, medians and interquartile ranges (IQR) were calculated. Boxplots show the median, lower and upper quartiles, and whiskers denoting 5% and 95% percentiles. Descriptive statistics included median age and sex distribution. Non‐parametric tests were used for group comparisons: Mann–Whitney *U* test for independent samples, Wilcoxon test for paired samples, and chi‐square test for categorical variables. A *p* value < 0.05 was considered statistically significant.

## 3 Results

### 3.1 Patient Characteristics

A total of 32 patients with PAD were included, comprising 21 patients with IC and 11 with CLTI. Baseline anthropometric characteristics, sex distribution, comorbidities and medication were comparable between groups. Among the CLTI cohort, eight out of 11 patients (72.7%) had pain at rest, and eight (72.7%) had ulcers or chronic wounds. Successful revascularisation was confirmed postoperatively by duplex ultrasound alongside improvements in walking distance and ABPI (IC: *p* = 0.001; CLTI: *p* = 0.046). All eight CLTI patients with wounds demonstrated postoperative improvement in wound healing, with seven achieving complete resolution. Further baseline characteristics are detailed in Table 1.

**TABLE 1:** Anthropometric and clinical parameters.

Anthropometric and clinical parameters			
	IC	CLTI	*p*
	(*n* = 21)	(*n* = 11)	IC/CLTI
Age (years)	68.0 (61.0–74.1)	70.2 (65.9–77.1)	0.351
Sex (%male)	18 (85.7)	10 (90.9)	0.673
Body mass index (BMI)	26.9 (20.2–29.6)	24.6 (20.1–30.8)	0.827
Diabetes	6 (28.6)	4 (36.4)	0.652
Type 1 (yes)	1 (4.8)	0	0.414
Active cigarette smoking	12 (57.1)	7 (63.6)	0.722
Former cigarette smoking	4 (19.0)	3 (27.3)	1.000
Pack years active smokers	50.0 (50.0–58.8)	55.0 (40.0–55.0)	0.751
Pack years former smokers	40.0 (40.0–55.0)	37.5 (15.0–37.5)	0.803
Hyperlipidemia	14 (66.7)	5 (45.5)	0.246
Any cardiovascular risk factor	19 (90.5)	9 (81.8)	0.482
Permanent dialysis	2 (9.5)	1 (9.1)	0.968
Aortoiliac PAD	6 (28.6)	2 (18.2)	0.519
Femoropopliteal PAD	19 (90.5)	11 (100.0)	0.290
Distal PAD	0	1 (9.1)	0.160
Technique of revascularisation			0.279
Open	8 (38.1)	6 (54.5)	
Endovascular	4 (19.0)	0	
Hybrid	9 (42.9)	5 (45.5)	
No. of cardiovascular risk factors	2.0 (1.0–3.0)	2.0 (1.0–3.0)	0.935
Statins	8 (38.1)	7 (63.6)	0.169
Antihypertensive drugs	17 (81.0)	7 (63.6)	0.283
Antiplatelet agents	18 (85.7)	8 (72.7)	0.371
Anticoagulation	5 (23.7)	5 (45.5)	0.210

PAD‐specific parameters					
	IC	CLTI	*p*		
	(*n* = 21)	(*n* = 11)	IC/CLTI	IC pre/post	CLTI pre/post
Ankle brachial index (ABPI)				0.001	0.046
Preoperative	0.65 (0.53–0.83)	0.60 (0.39–0.84)	0.588		
After revascularisation	1.0 (0.96–1.00)	0.90 (0.83–1.00)	0.171		
Rutherford classification					
0	0	0			
1	0	0			
2	8 (38)	0			
3	13 (62)	0			
4	0	4 (36)			
5	0	6 (55)			
6	0	1 (9)			
Walking distance				< 0.001	0.015
Improvement (yes)	18 (94.7)	9 (100)	0.491		
Walking distance without limitations (yes)	16 (84.2)	7 (77.8)	0.684		

Characteristics of CTLI (*n* = 11)					
Pain at rest		3 (27)			
Non‐healing wounds/ulcers/tissue lost		3 (27)			
Pain at rest and non‐healing wounds		5 (45)			
SVS WIfI score	Wound	Ischemia	Foot infection		
Patient 1	3	3	3		
Patient 2	2	2	2		
Patient 3	3	2	1		
Patient 4	3	1	1		
Patient 5	0		0		
Patient 6	0		0		
Patient 7	0	1	0		
Patient 8	3		2		
Patient 9	0	1	0		
Patient 10	3		0		
Patient 11	3	3	3		

*Note:* Cardiovascular risk factors included hypertonus, smoking status, diabetes mellitus and hyperlipidemia; Society for Vascular Surgery Lower Extremity Threatened Limb (SVS WIfI) classification system which stands for Wound Ischemia foot Infection defines CLTI patients, each factor is divided into four levels 0–3 (none–mild–moderate–severe) [18]; frequencies are presented as prevalences with percentages in brackets; continuous data are expressed as median with interquartile range in brackets. Statistical analysis by chi squared, Mann–Whitney test and Wilcoxon test for the pre‐ and postoperative comparisons. A *p* value < 0.05 was considered statistically significant.

Abbreviations: ABPI, ankel brachial index; CLTI, chronic limb threatening ischemia; IC, intermittent claudication; PAD, peripheral arterial disease.

### 3.2 Mitochondrial Content Marker—Citrate Synthase Activity (CSA)

Postoperatively, CSA increased in the ischemic gastrocnemius muscle of the CTLI group (*p* = 0.043). No significant changes in CSA were observed in the vastus lateralis muscle in either group (Table 2 and Supporting Information S2).

**TABLE 2:** Citrate synthase activity.

Muscle	*N*	CSA [nmol/min/mg protein]		*p*
		Preoperative	Postoperative	
Gastrocnemius muscle				
IC	18	344.5 (243.7–433.9)	391.6 (285.1–471.5)	0.267
CLTI	7	355.4 (260.0–401.1)	399.7 (334.3–512.4)	**0.043**
Vastus lateralis muscle				
IC	12	300.0 (236.4–355.6)	305.4 (179.4–368.2)	0.937
CLTI	6	272.4 (139.2–312.2)	226.5 (167.8–305.0)	0.917

*Note:* Citrate synthase activity (CSA) in [nmol/min/mg protein] of gastrocnemius and vastus lateralis muscles in patients with intermittent claudication (IC) and in chronic limb threatening ischemia (CLTI) preoperative and 6 weeks after successful revascularisation. Data are expressed as median with interquartile range in brackets. Statistical analysis by Wilcoxon test. A *p* value < 0.05 was considered statistically significant.

### 3.3 Mitochondrial Respiration—HRR

In both the gastrocnemius muscle and vastus lateralis muscle, mitochondrial respiration showed no significant pre‐ to postoperative differences across any respiratory state in either group (Table 3).

**TABLE 3:** Mitochondrial respiration.

(a) GM–mitochondrial respiration [pmol/(s*mg)]														
	IC					CLTI					*p*			
	*N*	Preoperative		Postoperative		*N*	Preoperative		Postoperative					
		*M*	IQR	*M*	IQR		*M*	IQR	*M*	IQR	IC pre/post	CLTI pre/post	IC/CLTI pre	IC/CLTI post
MOct_L_	19	13.0	11.0–15.4	12.7	10.6–17.8	11	10.5	6.8–16.2	15.0	7.8–18.8	0.809	0.248	0.478	0.931
MOct_P_	19	23.8	16.1–29.8	22.8	16.6–28.7	10	22.4	6.9–29.0	24.0	17.1–32.9	0.872	0.445	0.435	0.748
P	20	66.6	49.7–78.1	57.5	44.4–65.2	11	55.1	19.7–64.6	53.0	24.0–74.8	0.126	0.929	0.063	0.528
ET	20	76.2	51.8–90.9	63.9	48.4–71.5	11	65.6	26.1–79.1	57.7	26.9–98.3	0.135	1.000	0.302	0.741
Rot	20	43.8	35.8–62.8	33.8	28.1–50.9	10	49.1	24.4–59.4	33.9	14.2–47.4	0.108	0.139	0.826	0.660

(b) VLM–mitochondrial respiration [pmol/(s*mg)]														
	IC					CLTI					*p*			
	*N*	preOP		postOP		*N*	preOP		postOP					
		*M*	IQR	*M*	IQR		*M*	IQR	*M*	IQR	IC pre/post	CLTI pre/post	IC/CLTI pre	IC/CLTI post
MOct_L_	15	17.9	10.5–20.2	15.0	10.2–19.7	11	14.6	10.1–25.2	17.9	11.1–19.8	0.460	0.722	0.938	0.551
MOct_P_	15	28.2	14.2–29.7	26.8	19.5–30.4	11	21.8	12.3–30.5	25.6	19.2–30.4	0.281	0.182	0.421	0.775
P	16	60.1	45.6–65.6	53.3	48.6–60.9	11	54.2	47.8–61.8	48.7	42.9–54.7	0.642	0.657	0.430	0.183
ET	16	69.1	49.8–106.0	61.7	53.7–78.1	11	64.8	52.5–93.6	58.8	49.4–80.5	0.179	1.000	0.521	0.730
Rot	15	48.4	39.1–59.7	39.8	35.1–43.8	10	48.6	30.0–62.8	40.8	32.5–50.1	0.156	0.445	0.912	0.912

*Note:* Mitochondrial respiration [pmol/(s*mg)] in (a) gastrocnemius muscle (GM) and (b) vastus lateralis muscle (VLM) preoperative and postoperative 6 weeks after successful revascularisation of intermittent claudication (IC) and chronic limb threatening ischemia (CLTI) patients. Data are presented as median with interquartile range (IQR). Statistical analysis by Wilcoxon test and Mann–Whitney test. A *p* value < 0.05 was considered statistically significant.

Abbreviations: ET, maximal respiration after addition of FCCP; MOct_L_, maximal respiration after titration of malate and octanoyl; MOct_P_, maximal respiration after addition of ADP; P, maximal respiration after addition of pyruvate and succinate; Rot, inhibition of Complex I.

### 3.4 Mitochondrial Respiration Normalised to CSA

In the gastrocnemius muscle of the IC group, mitochondrial respiration per CSA significantly declined postoperatively for the following parameters: maximal ADP‐stimulated respiration (MOct_P_, *p* = 0.001), and complex II after inhibition of complex I (Rot, *p* = 0.031). No such postoperative changes were observed in the CLTI group. Additionally, a significant intergroup difference in electron transport capacity (ET) was noted postoperatively (*p* = 0.045) (Table 4a; Figure 1). In the vastus lateralis muscle, no significant pre‐ to postoperative changes were observed in any respiratory state normalised to CSA in either group (Table 4b).

**TABLE 4:** Mitochondrial respiration normalised to citrate synthase activity.

(a) GM–Mitochondrial respiration [pmol/(s*mg)] per CSA														
	IC					CLTI					*p*			
	*N*	Preoperative		Postoperative		*N*	Preoperative		Postoperative					
		*M*	IQR	*M*	IQR		*M*	IQR	*M*	IQR	IC pre/post	CLTI pre/post	IC/CLTI pre	IC/CLTI post
MOct_L_	16	0.037	0.028–0.064	0.035	0.030–0.040	7	0.032	0.016–0.049	0.023	0.018–0.041	0.255	0.398	0.285	0.161
MOct_P_	16	0.064	0.052–0.115	0.054	0.049–0.079	6	0.044	0.026–0.097	0.050	0.015–0.071	**0.001**	0.463	0.210	0.376
P	17	0.205	0.140–0.260	0.136	0.118–0.172	7	0.178	0.079–0.200	0.103	0.047–0.183	0.084	0.063	0.105	0.070
ET	17	0.212	0.150–0.273	0.140	0.125–0.183	7	0.185	0.097–0.213	0.110	0.047–0.217	0.124	0.128	0.240	**0.045**
Rot	17	0.153	0.094–0.184	0.086	0.068–0.122	7	0.119	0.044–0.0162	0.065	0.027–0.104	**0.031**	0.063	0.216	0.081

(b) VLM–mitochondrial respiration [pmol/(s*mg)] per CSA														
	IC					CLTI					*p*			
	*N*	preOP		postOP		*N*	preOP		postOP					
		*M*	IQR	*M*	IQR		*M*	IQR	*M*	IQR	IC pre/post	CLTI pre/post	IC/CLTI pre	IC/CLTI post
MOct_L_	10	0.047	0.035–0.058	0.054	0.046–0.062	6	0.047	0.027–0.146	0.061	0.033–0.103	0.646	0.753	0.914	0.828
MOct_P_	10	0.078	0.047–0.099	0.090	0.074–0.115	6	0.067	0.046–0.156	0.100	0.041–0.165	0.508	0.116	1.000	0.914
P	11	0.179	0.146–0.271	0.184	0.155–0.289	6	0.220	0.142–0.392	0.230	0.157–0.281	0.929	0.600	0.421	0.615
ET	11	0.190	0.162–0.246	0.243	0.173–0.326	6	0.230	0.155–0.549	0.272	0.168–0.381	0.722	0.463	0.481	0.615
Rot	11	0.139	0.116–0.162	0.148	0.096–0.208	5	0.154	0.089–0.345	0.155	0.095–0.254	0.929	0.500	0.865	0.692

*Note:* Mitochondrial respiration normalised to citrate synthase activity [pmol/(s*mg)/CSA] in (a) gastrocnemius muscle (GM) and (b) vastus lateralis muscle (VLM) preoperative and postoperative 6 weeks after successful revascularisation of intermittent claudication (IC) and chronic limb threatening ischemia (CLTI) patients. Data are presented as median with interquartile range (IQR). Statistical analysis by Wilcoxon test and Mann–Whitney test. A *p* value < 0.05 was considered statistically significant.

Abbreviations: ET, maximal respiration after addition of FCCP; MOct_L_, maximal respiration after titration of malate and octanoyl; MOct_P_, maximal respiration after addition of ADP; P, maximal respiration after addition of pyruvate and succinate; Rot, inhibition of Complex I.

**FIGURE 1:**
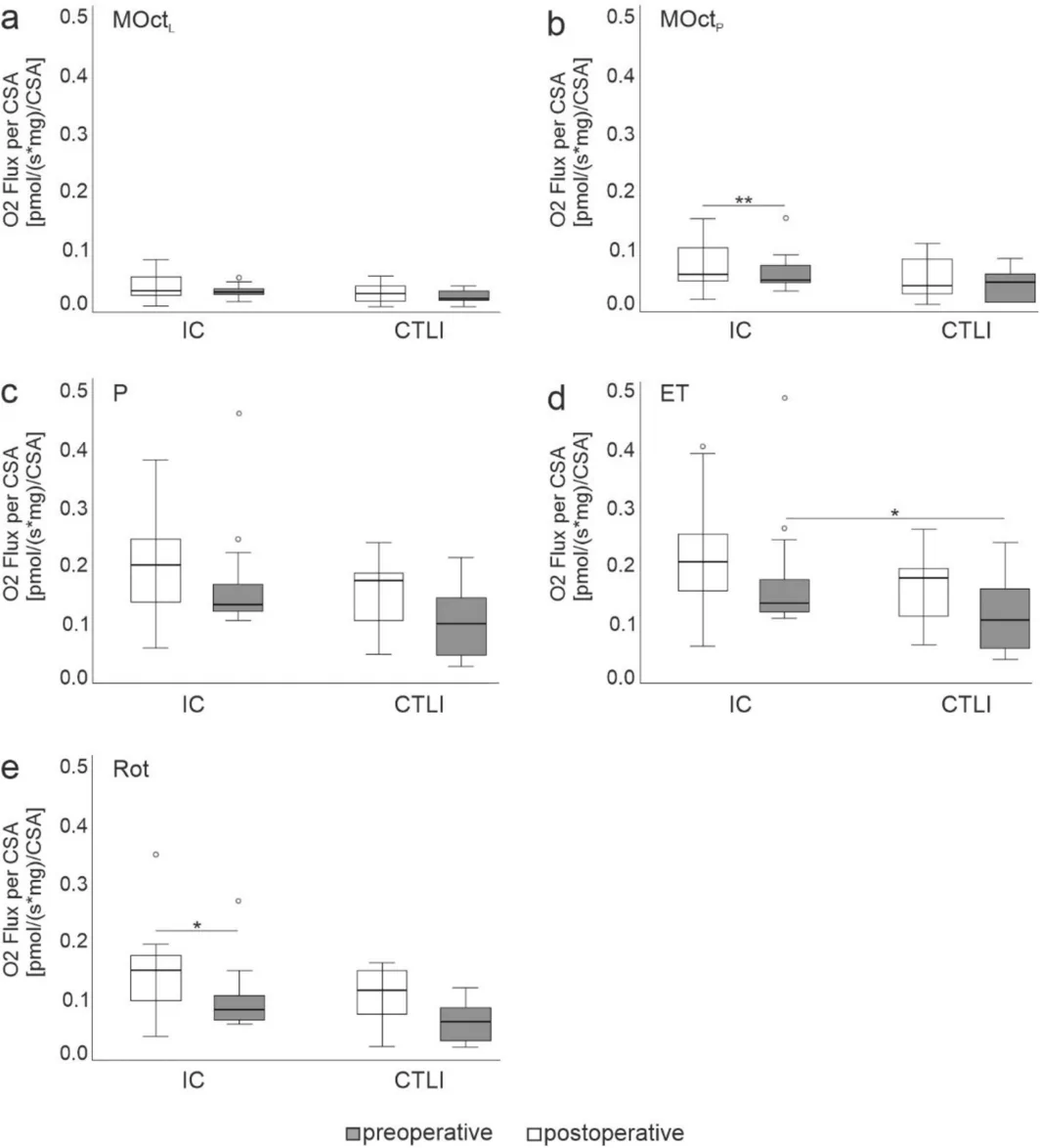
Mitochondrial respiration of gastrocnemius muscle normalised to citrate synthase activity [pmol/(s*mg)/CSA] from intermittent claudication (IC) and chronic limb threatening ischemia (CLTI). (a) MOct_L_—maximal respiration after titration of malate and octanoyl; (b) MOct_P_—maximal respiration after addition of ADP; (c) P—maximal respiration after addition of pyruvate and succinate; (d) ET—maximal respiration after addition of FCCP; (e) Rot—inhibitor of Complex I showed respiration of Complex II. *p < 0.05, **p < 0.01.

### 3.5 Gastrocnemius‐to‐Vastus Lateralis (GV) Respiration Ratio

GV ratio of mitochondrial respiration was significantly reduced in the IC group postoperatively for MOct_P_ (*p* = 0.022), OXPHOS capacity (P) (*p* = 0.003), ET (*p* = 0.004) and Rot (*p* = 0.013). No significant changes were observed in the CLTI group after revascularisation. CSA values remained unchanged postoperatively in both groups (Table 5 and Figure 2).

**TABLE 5:** Ratio of respiration in gastrocnemius over vastus muscle ‘GV‐ratio’.

	IC					CLTI					*p*			
	*N*	preOP		postOP		*N*	preOP		postOP					
		*M*	IQR	*M*	IQR		*M*	IQR	*M*	IQR	IC pre/post	CLTI pre/post	IC/CLTI pre	IC/CLTI post
CSA	11	1.238	0.969–1.575	1.245	1.072–1.537	4	1.008	0.714–1.521	1.301	0.988–1.774	0.594	0.465	0.296	0.896
MOct_L_	10	0.987	0.860–1.214	0.909	0.765–1.080	4	0.884	0.600–1.852	0.684	0.494–1.593	0.169	0.465	0.671	0.396
MOct_P_	10	1.050	0.865–1.329	0.875	0.720–1.066	4	0.790	0.644–1.189	1.265	0.582–1.456	**0.022**	0.465	0.157	0.229
P	11	1.136	0.970–1.428	0.872	0.801–1.080	4	1.063	0.685–1.086	1.380	0.475–1.817	**0.003**	0.465	0.090	0.151
ET	11	1.079	0.927–1.327	0.854	0.711–0.970	4	1.035	0.748–1.131	1.405	0.538–1.973	**0.004**	0.465	0.433	0.240
Rot	10	1.063	0.893–1.363	0.728	0.657–0.910	4	0.986	0.534–1.253	1.047	0.382–1.090	**0.013**	0.465	0.480	0.203

*Note:* Ratio of mitochondrial respiration in the gastrocnemius to vastus muscle (GV‐ratio) in patients with intermittent claudication (IC) and chronic limb threatening ischemia (CLTI), assessed preoperatively and 6 weeks postoperatively following successful revascularisation. CSA citrate synthase activity [nmol/min/mg protein]; mitochondrial respiration in [pmol/(s*mg)]. Data are presented as median with interquartile range (IQR). Statistical analysis by Wilcoxon test and Mann–Whitney test. A *p* value < 0.05 was considered statistically significant.

Abbreviations: ET, maximal respiration after addition of FCCP; MOct_L_, maximal respiration after titration of malate and octanoyl; MOct_P_, maximal respiration after addition of ADP; P, maximal respiration after addition of pyruvate and succinate; Rot, inhibition of Complex I. A *p* value 〈 0.05 was considered statistically significant.

**FIGURE 2:**
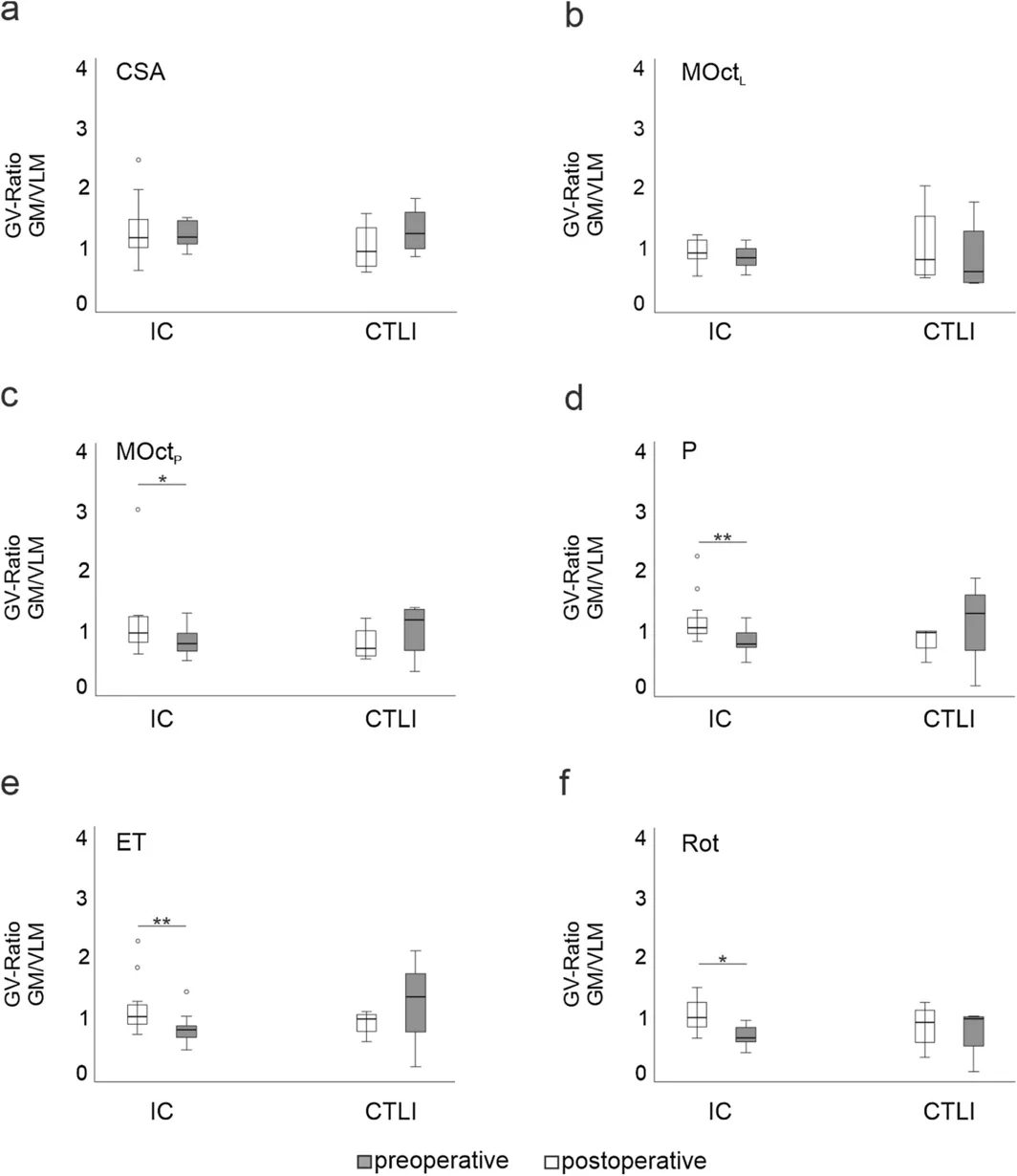
Ratio of respiration in gastrocnemius over vastus muscle ‘GV‐ratio’. Ratio of respiration in gastrocnemius (GM) over vastus muscle (VLM) ‘GV‐ratio’ in intermittent claudication (IC) and chronic limb threatening ischemia (CLTI), for mitochondrial content [nmol/min/mg protein] and all respiratory states [pmol/(s*mg)] defined. (a) CSA citrate synthase activity; (b) MOct_L_—maximal respiration after titration of malate and octanoyl; (c) MOct_P_—maximal respiration after addition of ADP; (d) P—maximal respiration after addition of pyruvate and succinate; (e) ET—maximal respiration after addition of FCCP; (f) Rot—inhibitor of Complex I showed respiration of Complex II. *p < 0.05, **p < 0.01.

## 4 Discussion

Adaption of mitochondrial respiration and content in skeletal muscle appears to be limited to milder stages of peripheral artery disease (PAD), such as intermittent claudication (IC) [15]. This study aimed to determine whether the severity of PAD influences the regenerative capacity of mitochondrial respiration and content following revascularisation. Our results indicate that mitochondrial adaptability after successful revascularisation is largely restricted to patients with less advanced disease stages, such as IC. In the context of an aging society and the increasing prevalence of atherosclerosis and stenotic lesions requiring intervention [2, 21, 22, 23], understanding disease patterns has become crucial for healthcare systems.

Six weeks after revascularisation, we observed an increase in mitochondrial content—determined by CSA—but only in the chronic limb‐threatening ischemia (CLTI) group of gastrocnemius muscle. Given that exercise training is known to enhance mitochondrial content [24, 25], and the CLTI group exhibited greater improvement in walking distance (Table 1), the absence of a statistically significant increase in mitochondrial content observed in IC patients may reflect lower exercise stimulus. In our previous studies we identified mitochondrial content differences in IC patients. However, the IC group was previously more homogeneous, as it consisted exclusively of isolated stenoses of the superficial femoral artery [16, 20]. As the heterogeneity of the groups increased, we recently observed that, compared to our control patients, CSA values differed only in the CLTI group [15]. Some studies reported no preoperative CSA differences between IC, CLTI, and control groups [6, 26], while others observed elevated CSA in IC patients compared to matched controls [27]. It is difficult to make a comparison here, as the postoperative values following revascularisation are missing for classification purposes. As well as Park et al. [27] included milder IC stages compared to our IC group. Pedersen et al. [26] compared CLTI patients before and after revascularisation and distinguished between CLTI patients with and without diabetes. They saw no differences of CSA values at all [26]. Since our study does not provide a further breakdown based on the presence of diabetes, comparability remains limited. Wilburn et al. [28] assessed CSA before and after revascularisation in IC patients and found no differences. Unlike our study, Wilburn et al. [28] chose a significantly longer postoperative follow‐up period (6 months versus 6 weeks). During this period, disease‐related changes associated with the systemic progression of peripheral arterial disease (PAD) could mask or alter the effects of revascularisation. A direct comparison of the results is therefore not possible.

More advanced disease states such as CLTI seem to exhibit limited adaptation of mitochondrial respiration [15]. In the present study, the IC group showed a postoperative decrease in mitochondrial‐specific complex I and II respiration, whereas the CLTI group demonstrated no significant changes across any respiratory state 6 weeks after revascularisation of gastrocnemius muscle. When compared to control patients from our previous study [15], preoperatively elevated complex I respiration normalised to CSA in the IC group decreased to control levels postoperatively suggesting normalisation. No such normalisation occurred in CLTI patients. These findings support our hypothesis that mitochondrial adaptive capacity following revascularisation is predominantly occurring in earlier stages of PAD, such as IC.

Ryan et al. [29] compared gastrocnemius muscle from CLTI patients at both interventional and amputation stages to controls and found profound mitochondrial dysfunction, in terms of downregulated mitochondrial proteome measured via LC–MS/MS for TMT (Liquid Chromatography–Tandem Mass Spectrometry) and impaired mitochondrial function of the fatty acid and carbohydrate pathway measured by HRR with creatinine‐kinase clamp system, in amputation‐stage patients. Many of whom had previously undergone revascularisation. This supports the notion that adaptive capacity diminishes with disease progression.

In contrast, Wilburn et al. [28] observed increased Complex I–IV respiration normalised to CSA 6 months post‐revascularisation in IC patients. However, their study employed a different SUIT protocol and a longer postoperative interval, limiting direct comparability. In our previous study [9], we observed decreased mitochondrial content and increased mitochondrial respiration per wet weight as well as normalised to CSA preoperatively, which normalised postoperatively. We interpreted this as evidence of adaptive capacity of mitochondrial functional, a finding reinforced by our current results. The lack of a control group and the extended postoperative interval in the study of Wilburn et al. [28] make comparability difficult. Distortion due to the progression of the disease during the extended postoperative interval is conceivable.

In the study by Pedersen et al. [26], only one—the succinate‐driven respiration—of five high‐resolution respirometry protocols showed a significant increased complex II respiration normalised to CSA 6 weeks postoperatively in diabetic CLTI patients. No differences were found between diabetic and non‐diabetic CLTI groups, either in mitochondrial respiration or CSA. Due to these similarities—and the absence of a difference in diabetes distribution between our IC and CLTI groups (Table 1), we did not stratify patients based on diabetes status, as done in other comparable studies [6, 28].

Due to high inter‐individual variability of HRR in PAD patients, we previously introduced the ratio of respiration in gastrocnemius muscle over vastus lateralis muscle (‘GV ratio’) as an intra‐individual normalisation tool [15]. In the present study, changes in the GV ratio post‐revascularisation further supported our hypothesis of a limited mitochondrial adaptability in advanced PAD (Table 5 and Figure 2). Specifically, IC patients demonstrated a decrease in respiration values postoperatively, approximating control levels from our previous study [15], while CLTI patients showed no significant changes, indicating an absence of mitochondrial adaptability.

The observed lack of mitochondrial adaptive capacity in CLTI patients compared to those with IC may be attributed to multiple factors. The absence of functional improvement following revascularisation in advanced PAD suggests a fundamental limitation in adaptive potential [30]. Additionally, the distinct mitochondrial dysfunction observed in CLTI [6], along with myopathy [31, 32] and neuropathy [33], indicates a more severe impairment. Mitochondriopathy leads to increased reactive oxygen species (ROS) production and consequently to increased oxidative stress. As mitochondriopathy increases, the threshold for ROS is exceeded and protective mitohormesis and antioxidant stimulation fail to occur [34]. In severe PAD stages, this could lead to a lack of adaptive capacity.

The more pronounced ischemia–reperfusion damage in CLTI further impairs mitochondrial function and reduces the potential for cellular repair and adaptation [35]. As training primarily enhances mitochondrial content rather than function [25, 36, 37], it cannot compensate for these deficits. Additionally, impaired microcirculation—which may be only partially reversible—may further limit tissue adaption in CLTI patients [38].

Omics analyses reveal significant differences of gene and protein expression and serum metabolites between CLTI, IC, and controls supporting that CLTI constitutes a distinct clinical entity with limited adaptive capacity [10, 11, 29, 39]. For example, Cong et al. [10] observed enriched fibrosis pathways in CLTI patients compared to controls and IC patients.

Since adequate reperfusion does not always translate into clinical improvement [30, 40], understanding the distinct biological responses in each PAD stage is vital for developing individualised treatment strategies. The delayed regenerative response in CLTI patients [26, 41], underscores the need for long‐term studies with extended postoperative follow‐up.

Schoenherr et al. [42] hypothesise in their study protocol that revascularisation (restored blood flow and oxygen delivery) would improve mitochondrial function more effectively than conservative exercise therapy and mitochondrial improvements in ischemic muscles would be more pronounced than in non‐ischemic controls. This randomised controlled trial aims to determine whether surgical revascularisation or exercise is more effective for mitochondrial adaptability in PAD—and correlate biochemical changes with functional outcomes—potentially shaping future personalised treatment paradigms.

One limitation of our study is the relatively small number of CLTI patients, especially regarding postoperative biopsies. However, comparable studies report similar sample sizes [26]. This may be due to the frailty of this population [43], which includes preoperative non‐healing wounds and a general reluctance to undergo repeated biopsy. Additionally, due to significant inter‐individual variability, paired analysis (Wilcoxon signed‐rank test) was required, limiting inclusion to those with complete CSA and HRR data both pre‐ and postoperatively. In some CLTI cases, biopsy samples were too small for full analysis.

Another limitation is the use of only one mitochondrial marker due to limited tissue availability. Moreover, we found no significant difference in ABPI between groups. In diabetic patients, ABPI may overestimate perfusion due to vascular calcification, and toe pressure—which would have been more reliable—was not assessed [44]. The lack of a matched control group without PAD, particularly in terms of age and comorbidities, should also be mentioned as a limitation. Furthermore, no information is available on physical activity patterns within the 6‐week period following revascularisation.

## 5 Conclusion

This study provides evidence for a stage‐dependent adaptive capacity of mitochondrial function in the skeletal muscle of PAD patients following successful revascularisation. Specifically, postoperative improvements in mitochondrial respiration were observed exclusively in patients with intermittent claudication, indicating that the adaptability of mitochondria appears to be largely confined to the earlier stages of PAD. In contrast, patients with chronic limb‐threatening ischemia exhibited no significant mitochondrial adaptation, suggesting that prolonged ischemic damage in advanced PAD stages limit the potential for adaptability. These findings underscore the importance of early diagnosis and timely intervention. However, further studies with larger cohorts and extended follow‐up periods are warranted to confirm these results and to better understand the mechanisms underlying impaired mitochondrial adaptive capacity in advanced PAD.

## Author Contributions

**Fiona Speichinger:** writing – original draft, formal analysis, data curation, resources, visualization, investigation, conceptualization. **Alexandra Gratl:** methodology, supervision, data curation, writing – review and editing, resources, conceptualization. **Ben Raude:** data curation. **Marco Arndt:** data curation. **Nina A. Hering:** writing – review and editing, methodology. **Andreas Greiner:** conceptualization, supervision, writing – review and editing. **Dominik Pesta:** methodology, supervision, writing – review and editing, resources, conceptualization. **Christian Schineis:** writing – review and editing. **Jan Paul Frese:** project administration, supervision, writing – review and editing, methodology, validation, resources, conceptualization, formal analysis.

## Funding

The authors have nothing to report.

## Ethics Statement

The institutional research committee (Charité's Ethics Committee, Universitätsmedizin Berlin; number of proposal EA4/139/16) approved the study.

## Consent

Written informed consent was obtained from all study participants. All authors consent for publication.

## Conflicts of Interest

The authors declare no conflicts of interest.

## Supporting information


**Data S1:** Medium.

**Data S2:** Citrate synthase activity.

## Data Availability

The dataset generated and analysed during the current study is available from the corresponding author upon reasonable request.
