# Supplementary material for: Chronic Limb‐Threatening Ischemia Shows No Adaptability of Mitochondrial Function After Revascularisation
Source: J Cell Mol Med. 2026 Oct 16;30(20):e71383. doi: 10.1111/jcmm.71383 (PMC13675601; doi:10.1111/jcmm.71383)
Supplement: Supplementary file 1 [file JCMM-30-e71383-s001.docx]

**Supplement 1**

BIOPS

10 mM Ca-EGTA buffer, 0.1µM free calcium, 20 mM imidazole, 20mM taurine, 50 mM 2-(N-morpholino)esthane-sulfonic acid hydrate, 0.5 mM dithiothreitol, 6.25mM MgCl₂, 5.77 ATP, 15 mM phosphocreatine (pH 7.1)

MiR06

110 mM D-sucrose, 60 mM K^+^-lactobionate, 0.5 mM EGTA, 3 mM MgCl₂, 20 mM taurine, 10 mM KH₂PO_4_, 20 mM HEPES, 1g/l bovine serum albumin, 280 U/ml catalase
