# Supplementary material for: Chronic Limb‐Threatening Ischemia Shows No Adaptability of Mitochondrial Function After Revascularisation
Source: J Cell Mol Med. 2026 Oct 16;30(20):e71383. doi: 10.1111/jcmm.71383 (PMC13675601; doi:10.1111/jcmm.71383)
Supplement: Supplementary file 2 [file JCMM-30-e71383-s002.docx]

**Supplement 2 - Citrate Synthase Activity**

**Supplement 2** – **Citrate synthase activity (CSA) in [nmol/min/mg protein]** of **a** gastrocnemius and **b** vastus lateralis muscles in patients with intermittent claudication (IC) and in chronic limb threatening ischemia (CLTI) preoperative and 6 weeks after successful revascularization (postoperative). Statistical analysis by Mann-Whitney test. *p<0.05.
